# History of the American Medical Association, from Its Organization up to January, 1855

**Published:** 1855-10

**Authors:** 


ART. V.—*History of the American Medical Association, from its Organization up to January*, 1855. By N. S. Davis, M. D., Professor of Prin. and Prac. of Medicine in Rush Medical College, etc., etc. To which is appended biographical notices, with portraits of the Presidents of the Association, and of the Author. Edited by Geo. W. Butler, M. D. Philadelphia: Lippincott, Grambo & Co. 1855.

This beautiful volume is a record of the origin and progress of that great national body, now recognized as the paramount authority in medical ethics. By this we do not mean that the book is a volume of transactions, for that would be a work of supererogation, but a truthful account of its inception in the Medical Society of the State of New York, of the first convention at the city of New York, of the opposition it met from parties, then as now opposed to any attempt to "guard the shepherds," or interfere with the selling of diplomas like so many patent rights, and, finally, of its steady progress, until it is now an acknowledged and honored authority.

Our crowded space in this number forbids any lengthened notice of this important book. We can only say, that originally published in the pages of our enterprising cotemporary, the New Jersey Medical Reporter, with beautifully engraved portraits of the presidents, it has been thought worthy of preservation in book form. It has been issued in very neat and handsome style, and we doubt not will meet with the large sale it deserves.

We presume it may be found at our bookstores, but if not, it may be ordered through them from the publishers.

